# Biotinidase deficiency: clinical and genetic studies of 38 Brazilian patients

**DOI:** 10.1186/s12881-014-0096-3

**Published:** 2014-09-01

**Authors:** Taciane Borsatto, Fernanda Sperb-Ludwig, Louise LC Pinto, Gisele R De Luca, Francisca L Carvalho, Carolina FM De Souza, Paula FV De Medeiros, Charles M Lourenço, Reinaldo LO Filho, Eurico C Neto, Pricila Bernardi, Sandra Leistner-Segal, Ida VD Schwartz

**Affiliations:** 1 Post Graduate Program in Genetics and Molecular Biology, Universidade Federal do Rio Grande do Sul (UFRGS), Porto Alegre, Brazil; 2 BRAIN Laboratory, Center for Experimental Research (CPE), Hospital de Clínicas de Porto Alegre (HCPA), Porto Alegre, Brazil; 3 Post Graduate Program in Medicine: Medical Sciences, UFRGS, Porto Alegre, Brazil; 4 Gene Therapy Center, CPE, HCPA, Porto Alegre, Brazil; 5 Hospital Infantil Joana de Gusmão, Florianópolis, Brazil; 6 Medical Genetics Service, Hospital de Clínicas de Porto Alegre (HCPA), Rua Ramiro Barcelos, Porto Alegre, 90035-903, Brazil; 7 Universidade Federal de Campina Grande, Campina Grande, Brazil; 8 Medical Genetics Service, Hospital das Clínicas de Ribeirão Preto, Universidade de São Paulo, São Paulo, Brazil; 9 Universidade Estadual de Ciências da Saúde de Alagoas, Maceió, Brazil; 10 CTN Diagnósticos, Porto Alegre, Brazil; 11 Hospital Universitário-Universidade Federal de Santa Catarina, Florianópolis, Brazil; 12 Department of Genetics, UFRGS, Porto Alegre, Brazil

**Keywords:** Low biotinidase, Genetic variants, Neonatal screening, Brazil

## Abstract

**Background:**

Biotinidase deficiency (BD) is an inborn error of metabolism in which some genetic variants correlate with the level of enzyme activity. Biotinidase activity, however, may be artifactually low due to enzyme lability, premature birth, and jaundice; this hinders both phenotypic classification and the decision to implement therapy. This study sought to characterize the clinical and genetic profile of a sample of Brazilian patients exhibiting reduced biotinidase activity.

**Methods:**

This observational, multicenter study used a convenience sampling strategy, with sequencing of exons 2, 3, and 4 of the *BTD* gene.

**Results:**

The sample comprised 38 individuals with biochemical phenotypes defined a priori on the basis of biotinidase activity in serum/plasma (2 with profound deficiency, 9 with partial deficiency, 15 heterozygous, 1 borderline between partial deficiency and heterozygosity, 2 borderline between heterozygous and normal) or dried blood spot sample (n = 9, all with unspecified deficiency). Most patients were from Southern Brazil (n = 29/38) and were identified by neonatal screening (n = 33/38). Parental consanguinity was reported in two cases. The most commonly found genetic variants were c.1330G > C (p.D444H), c.755A > G (p.D252G), and c.[511G > A;1330G > C] (p.[A171T;D444H]), with allele frequencies of 50%, 9.4%, and 5.4% respectively. Three novel pathogenic variants were identified (c.119 T > C or p.L40P, c.479G > A or p.C160Y, and c.664G > A or p.D222N). Twenty-nine patients had two pathogenic variants detected (with *cis/trans* status ascertained in 26/29), six had only one variant, and three had no pathogenic variants detected. Genotyping confirmed the original phenotypic classification based on enzyme activity in 16/26 cases. Three polymorphic variants were identified in control individuals, of which two were nonpathogenic (c.1171C > T or p.P391S and c.1413 T > C or p.C471C, with a frequency of 1.5% and 5.5% respectively) and one pathogenic (c.1330G > C, frequency 4%).

**Conclusions:**

Our findings suggest that partial BD is the most common form of BD in Brazil, and expand current knowledge on the allelic heterogeneity of this condition.

## Background

Biotinidase deficiency (BD, EC 3.5.1.12) is an autosomal recessive disease in which both the absorption of biotin from dietary sources and its reuse/recycling are impaired. This leads to a deficiency in biotin-dependent enzymes, such as propionyl-CoA carboxylase (EC 6.4.1.3), β-methylcrotonyl-CoA carboxylase (EC 6.4.1.4), pyruvate carboxylase (EC 6.4.1.1), and acetyl-CoA carboxylase (EC 6.4.1.2) [1],[2]. Treatment, which consists of oral administration of free biotin, is simple and effective when started early (e.g., before the onset of symptoms). If treatment is delayed, there is a risk of irreversible damage (including hearing and vision loss and mental retardation) and even death [3]–[5]. The diagnosis of BD is confirmed by measurement of biotinidase activity in plasma or serum. This test enables classification of patients as having profound (or total) BD (residual activity <10%) or partial BD (activity within 10-30% of average normal activity in serum). Heterozygous individuals have enzyme activity levels between those of affected patients and those of homozygous normal individuals [2]. This classification plays an important role in instituting therapy, as patients with profound BD should be treated and heterozygous individuals should not; there is no consensus on the need for treatment of patients with partial BD [6].

However, enzyme testing is subject to interfering factors that can produce artifactually low results, such as the lability of biotinidase [7] and the direct correlation between enzyme activity and gestational age and even with postnatal age [8]. Furthermore, jaundiced neonates may have reduced biotinidase activity [9]. Analysis of the *BTD* gene is important, particularly to elucidate the diagnosis when repeated enzyme testing yields discordant results [10], in the assessment of premature infants with reduced biotinidase activity, and in the assessment of infants who have received blood transfusions [6]. Table 1 provides a review of what is known from the literature about the association between the biochemical phenotype and genotype in BD.

**Table 1:** Molecular basis of biotinidase deficiency (BD)

**BD**		**Unaffected individuals**			
**Profound BD**	**Partial BD**	**≈ Hz**	**Hz**	**≈ N**	**N**
Profound deficiency allele	Profound deficiency allele	p.D444H	Profound deficiency allele	p.D444H	Wild-type allele
+	+	+	+	+	+
Profound deficiency allele	p.D444H	p.D444H	Wild-type allele	Wild-type allele	Wild-type allele

Most variants in the *BTD* gene cause complete or almost complete loss of the biotinidase enzyme activity; they are called profound deficiency alleles (e.g., c.98_104del7ins3, c.1612C > T (p.R538C), c.1368A > C (p.Q456H), c.[511G > A;1330G > C] (p.[A171T;D444H]). The presence of two such alleles, in homozygous or compound heterozygous form, results in profound BD. Individuals who are compound heterozygotes for the c.1330G > C (p.D444H) variant and a profound deficiency allele are expected to have ~ 20-25% of the normal biotinidase activity. It is expected that individuals homozygous for the p.D444H variant have similar activity to individuals heterozygous (Hz) for profound deficiency alleles. Hz for p.D444H variant show similar activity to individuals homozygous for the wild-type allele (normal activity, N). The p.D444H variant in *cis* with a mutation that causes profound deficiency results in a profound deficiency allele [11]–[13].

This study seeks to describe the clinical and genetic profile of a sample of Brazilian patients presenting reduced biotinidase activity.

## Methods

This is a multicenter, observational, cross-sectional study with a convenience sampling strategy. The study protocol was approved by the ethics committee of Hospital de Clínicas de Porto Alegre (HCPA), and all patients and/or their legal guardians provided written informed consent.

The study sample comprised 38 unrelated subjects (21 male), aged 1 month to 18 years, who were recruited from several regions of Brazil (29 from the South, 3 from the Southeast, and 6 from the Northeast) during 2012–2013 by contacting Brazilian medical geneticists throughout the mailing list of the Brazilian Medical Genetics Society. The criterion for inclusion was reduced biotinidase activity (below the lower reference limit of the testing laboratory). Clinical variables such as age, biotinidase activity, and symptoms were obtained by a review of medical records and by a data collection form filled out by the referring physicians. For genetic analysis, blood was collected from patients and their parents into EDTA-containing tubes. Overall, samples from both parents were available for 27 patients, samples from only one parent were available for 6 patients, and 5 patients had no parental samples available. Anonymous samples from 100 healthy, adult controls from Southern Brazil were tested for the c.1330G > C (p.D444H) variant and for all novel variants first described in the present study.

### Biotinidase activity

Patients had undergone enzyme activity testing at four different laboratories (A, B, C, and D), one of which (laboratory A, n = 9/38 patients) only carries out filter-paper testing, a technique that cannot quantitate the degree of BD and expresses activity in units (U) (Neonatal Biotinidase kit, PerkinElmer®, Wallac Oy, Turku, Finland). In these cases, patients were diagnosed by the attending staff as having BD after three consecutive abnormal tests (below the 70U cutoff), and were thus not classified into partial or profound BD. The activity presented in this study is the average of the three tests (Table 2). The remaining patients (n = 29/38) underwent quantitative enzymatic testing (after abnormal neonatal screening or clinical suspicion) at laboratories B, C, and D, in plasma or serum, as described by Wolf et al. (1983) [2]. The normal reference range is 5.0–10 nmol/min/mL. The following enzyme activity ranges were used for classification of biochemical phenotype: <0.75, profound deficiency; 0.75–2.25, partial deficiency; 2.26–4.9, heterozygosity. Values within ± 0.1 of a cutoff point were classified as borderline. When more than one measurement was available, the highest level was used for classification (Table 2).

**Table 2:** Biochemical and molecular profile of patients with reduced biotinidase activity (n = 38)

**Patient**	**Gender**	**Allele 1**	**Allele 2**	**Expected type of BD according to genotype**	**Type of BD according to enzyme activity**^**b**^	**Biotinidase activity**^**a**^	**Manner of diagnosis**
01	F	c.643C > T (p.L215F)	c.755A > G (p.D252G)	Profound	N/A	21.88 U	NS
02^c^	M	c.755A > G (p.D252G)	c.755A > G (p.D252G)	Profound	Profound	0.1; 0.4; 0.33; 0.44; 0.16; 0.32	S
03	F	c.1612C > T (p.R538C)	c.1612C > T (p.R538C)	Profound	Profound	0.12	S
04	M	c.1330G > C (p.D444H)	c.98_104del7ins3	Partial	Partial	1.5	NS
05	F	c.1330G > C (p.D444H)	c.[470G > A;1330G > C] (p.[R157H;D444H])	Partial	Partial	1.8	NS
06	M	c.1330G > C (p.D444H)	c.[511G > A;1330G > C] (p.[A171T;D444H])	Partial	Partial	1.4	NS
07	F	c.1330G > C (p.D444H)	c.[511G > A;1330G > C] (p.[A171T;D444H])	Partial	Hz	2.5	NS
08	F	c.1330G > C (p.D444H)	c.[511G > A;1330G > C] (p.[A171T;D444H])	Partial	N/A	45.81 U	NS
09	F	c.1330G > C (p.D444H)	c.594_596delCGT (p.V199del)	Partial	Partial	1.2; 1.9	NS
10	F	c.1330G > C (p.D444H)	c.[595G > A;*1413 T > C*] (p.[V199M;*C471C*])	Partial	Hz	2.8	NS
11	M	c.1330G > C (p.D444H)	c.755A > G (p.D252G)	Partial	Partial	1.7	NS
12	F	c.1330G > C (p.D444H)	c.755A > G (p.D252G)	Partial	Partial	1.2	NS
13	F	c.1330G > C (p.D444H)	c.755A > G (p.D252G)	Partial	Hz	2.4	NS
14	M	c.1330G > C (p.D444H)	c.755A > G (p.D252G)	Partial	N/A	51.37 U	NS
15	M	c.1330G > C (p.D444H)	c.933delT	Partial	Partial	1.6	NS
16	F	c.1330G > C (p.D444H)^d^	c.100G > A^d^	Partial or Hz	Partial	1.2; 2.04	NS
17	M	c.1330G > C (p.D444H)^d^	c.643C > T (p.L215F)^d^	Partial or Hz	Hz	2.4	NS
18	F	c.1330G > C (p.D444H)^d^	c.1629C > A (p.D543E)^d^	Partial or Hz	Hz	2.6	NS
19	M	*c.1413 T > C (p.C471C)*	c.[511G > A;1330G > C] (p.[A171T;D444H])	Hz	Hz	3.3	NS
20	M	c.1595C > T (p.T532M)	N	Hz	Hz	1.5; 2.9; 4.4	NS
21	M	c.1330G > C (p.D444H)	c.1330G > C (p.D444H)	≈ Hz	Hz	0.7; 3.3	NS
22	M	c.1330G > C (p.D444H)	c.1330G > C (p.D444H)	≈ Hz	Hz	3.7	NS
23	M	c.1330G > C (p.D444H)	c.1330G > C (p.D444H)	≈ Hz	Hz	2.8; 2.7	NS
24	M	c.1330G > C (p.D444H)	c.1330G > C (p.D444H)	≈ Hz	N/A	48.75 U	NS
25	M	c.1330G > C (p.D444H)	c.1330G > C (p.D444H)	≈ Hz	N/A	51.83 U	NS
26	M	c.1330G > C (p.D444H)	c.1330G > C (p.D444H)	≈ Hz	N/A	46.88 U	NS
27^c^	M	c.1330G > C (p.D444H)	c.1330G > C (p.D444H)	≈ Hz	N/A	52.73 U	NS
28	F	c.1330G > C (p.D444H)	c.1330G > C (p.D444H)	≈ Hz	N/A	40.94 U	NS
29	F	c.1330G > C (p.D444H)	c.1330G > C (p.D444H)	≈ Hz	N/A	45.37 U	NS
30	M	c.1330G > C(p.D444H)	N	≈ N	Borderline (Hz/N)	4.9	NS
31	F	c.1330G > C (p.D444H)	N	≈ N	Hz	3.1	NS
32	F	c.1330G > C (p.D444H)	N	≈ N	Hz	3.8; 2.7	S
33	M	*c.1413 T > C (p.C471C)*	N	N	Borderline (Hz/N)	0.1; 2.6; 4.9	NS
34	F	N	N	N	Hz	4.1	S
35	M	N	N	N	Hz	3.7	S
36	F	c.1330G > C (p.D444H)	**c.119 T > C (p.L40P)**	Unknown	Partial	1.7	NS
37	M	c.1330G > C (p.D444H)	**c.479G > A (p.C160Y)**	Unknown	Borderline (Partial/Hz)	0.2; 1.7; 2.2	NS
38	M	**c.664G > A (p.D222N)**	N	Unknown	Hz	3.5	NS

^a^The cutoff for filter-paper tests performed at laboratory A is 70U. For quantitative testing in serum or plasma performed at laboratories B, C, D, the measurement unit is nmol/min/mL and the reference range is 5.0-10. Unless otherwise specified, the unit of enzyme activity is nmol/min/mL.

^b^The following enzyme activity ranges were used for phenotypic classification: <0.75, profound deficiency; 0.75-2.25, partial deficiency; 2.26-5.0, heterozygosity. If more than one measurement was obtained, the highest value was considered. Values within ± 0.1 of a cutoff point were classified as borderline.

^c^Patients with consanguineous parents.

^d^Whether it is in *cis* or *trans* configuration with the other variant found remains undetermined.

Novel variants shown in bold type, and synonymous variants, in italics.

BD, biotinidase deficiency; F, female; M, male; NS, neonatal screening; S, symptoms; Hz, heterozygosity; N/A, not available; N, normal.

### *BTD* gene analysis

Genomic DNA (gDNA) was extracted from blood collected into EDTA-containing tubes using an Easy-DNA™ Kit (Invitrogen™, Carlsbad, CA, USA) in accordance with manufacturer instructions. Exons 2, 3, and 4 and exon/intron junctions of the *BTD* gene were amplified and sequenced. Regions with detectable changes were assessed in the patients’ parents (data not shown), to confirm whether variants were in *cis* or *trans*. Polymerase chain reaction (PCR) was performed using previously described primers [14] and the following annealing temperatures: 58°C for amplicons 2 and 3, 59°C for 4a, 60°C for 4b, and 62°C for 4c and 4d. PCR products were purified with polyethylene glycol 8000 solution (PEG 8000 50%, NaCl 2.5 M) and sequenced using a BigDye® Terminator v3.1 Cycle Sequencing Kit and ABI 3500 Genetic Analyzer (Applied Biosystems, Foster City, CA, USA). Finally, the resulting sequences were compared with the reference sequence of the *BTD* gene (NG_008019.1).

For calculation of allele frequencies, the total number of alleles was set at 74, as two patients were the children of consanguineous parents.

A hundred control subjects were tested for the presence of variants c.119 T > C (p.L40P) and c.664G > A (p.D222N) by restriction fragment length polymorphism (RFLP) analysis – allele C of c.119 T > C introduces a site for the restriction enzyme *SmaI*, whereas allele A of c.664G > A removes a cleavage site for *TaqI* – and variants c.479G > A (p.C160Y) and p.D444H by direct sequencing of amplicons 4a and 4d respectively, which enabled identification of additional variants in these amplicons.

### *In silico* analysis

All variants described in patients for the first time in this study were evaluated for pathogenicity by *in silico* analysis with PolyPhen-2 [15] and SIFT [16] softwares.

### Genotype-phenotype association

Expected biochemical phenotype (e.g., profound DB, partial DB or heterozygous state) was established according to Table 1 only for genotypes composed of recurrent pathogenic variants with a known *cis/trans* configuration, and that are known to be associated with the phenotype.

## Results

### Clinical aspects

Of the 38 subjects included in the study (Table 2), only two had profound BD. Nine patients had undergone filter-paper screening alone, and thus had unspecified BD. Two patients (5.3%, patients 02 and 27) had consanguineous parents. Only one subject (patient 03) had a family history suggestive of BD recurrence – namely, a sister who died at age 3 years with clinical manifestations consistent with BD, but no confirmed diagnosis.

Thirty-three patients were identified by neonatal screening, of whom 27 were on biotin (10 mg/day); none had clinical manifestations suggestive of BD at the time of inclusion. Six patients (patients 06, 12, 15, 19, 22, 33) were preterm and had been born between 35 and 36 weeks of pregnancy. Neonatal jaundice was reported for patients 20 and 33. Another five patients had been diagnosed on the basis of clinical suspicion. In these patients, the most common symptoms were visual disturbances, neurological manifestations, and skin lesions. The age at onset of manifestations ranged from 1 day to 10 years, and the age at diagnosis from 40 days to 18 years. All were receiving biotin supplementation (10–20 mg/day) at the time of writing.

### Genetic assessment

A total of 17 different alleles were found among the analyzed patients (Table 2): 13 recurrent pathogenic variants, three novel variants (p.L40P, p.C160Y and p.D222N), and one synonymous variant. No changes were found in exon 3. The most common variants were p.D444H, c.755A > G (p.D252G) and p.[A171T;D444H], with allele frequencies of 50%, 9.4%, and 5.4% respectively.

Twenty-nine patients had two pathogenic variants detected (with *cis/trans* status ascertained in 26/29), six had only one variant, and three had no pathogenic variants detected. Among patients in whom both classification of biochemical phenotype and genotyping were possible (n = 26/38), genotyping confirmed the original classification in 16. Discordance between genotype and biochemical phenotype was seen in seven cases: patients 07, 10 and 13 exhibited enzyme activity levels slightly higher (suggestive of heterozygosity) than expected in view of their genotype (suggestive of partial BD); and patients 31, 32, 34 and 35 showed enzyme activity lower (suggestive of heterozygosity) than expected in view of the genotype (no variants found). In three subjects (patients 16, 17 and 18), the unclear *cis/trans* status prevented the confirmation of the biochemical phenotype. In patients 36, 37 and 38, genotyping revealed novel variants, thus precluding prediction of biochemical phenotype.

None of the three novel variants described herein were found in controls and two out of them were predicted as damaging by *in silico* analysis as well. The p.D222N variant had contradictory prediction between the two programs used (damaging by PolyPhen-2 and tolerated by SIFT). The allele frequency of p.D444H in controls was 4% (six heterozygous and one homozygous individuals for this variant were detected). Other variants found in controls were c.460-7_8insT, c.1171C > T (p.P391S), c.1284C > T (p.Y428Y) and c.1413 T > C (p.C471C) and their frequencies were respectively 0.5%, 1.5%, 0.5% and 5.5%.

## Discussion

This study characterized the clinical and molecular profile of Brazilian patients with reduced biotinidase activity and, thus, at risk of BD. Before 2012, neonatal screening for BD was unavailable through the public Unified Health System in the majority of Brazilian states, and was only provided by private laboratories located mainly in the South and Southeast regions of the country, which may have contributed to its underdiagnosis. Paradoxically, studies based on neonatal screening suggest that the overall frequency of BD (profound + partial BD) in Brazil may be among the highest ever reported, ranging from 1:6,843 to 1:62,500 newborns (NB) [17]–[20]. To the best of our knowledge, only one prior study conducted DNA analysis of Brazilian patients with BD (n = 21) [18].

Two studies conducted in the U.S., in which 92 patients with profound BD and 19 with partial BD were assessed, showed that the most common *BTD* variants in the study population were c.98_104del7ins3, c.1612C > T (p.R538C), c.1368A > C (p.Q456H), p.[A171T;D444H] and p.D444H, which, overall, accounted for approximately 60% of abnormal alleles found in the sample [12],[21]. Taking into account this five-variant panel, p.[A171T;D444H] and p.D444H account for approximately 55% of alleles detected in the present study, with the occurrence of variants c.98_104del7ins3 and p.R538C in only one patient each. In our sample, we did not detect the variant p.Q456H, the profound BD allele most commonly found among children diagnosed by neonatal screening in the U.S. (allele frequency = 28%) [21], nor did we find any changes in exon 3. Furthermore, p.D252G appeared to occur more commonly (allele frequency = 9.4%) in our sample than in the U.S. population [21]. Our findings corroborate those of Neto et al. (2004) [18], who analyzed the *BTD* gene in 21 Brazilian patients with reduced biotinidase activity detected by neonatal screening and also found that p.D444H, c.98_104del7ins3, p.[A171T;D444H], and p.D252G were the most prevalent variants; the authors also did not detect variant p.Q456H or changes in exon 3 among their sample. Therefore, we suggest that the profile of genetic variants found among Brazilian patients with BD differs from that found in U.S. patients, although these differences may be partly attributable to the relatively small number of patients with profound BD included in our sample (c.98_104del7ins3, p.R538C and p.Q546H are classically associated with profound BD).

Since the p.D444H variant - usually classified as a partial DB allele - was the most common variant found in our study, our findings also suggest that partial BD is the most common form of BD in Brazil. We found that allele C of the variant c.1330G > C or p.D444H constituted 4% of alleles in healthy individuals, a frequency close to that found in the U.S. population [22],[23], where the incidence of profound and partial BD has been estimated at 1:80,000 NB and 1:31,000–1:40,000 NB respectively [10], and in Western Hungary, where the incidence of profound and partial BD is 1:97,000 NB and 1:23,000 NB respectively [24].

As previously reported, the association between *BTD* genotype and biotinidase activity is not absolute [18],[25]. In the Greek study conducted by Thodi et al. (2013) [25], for instance, homozygosity for p.D444H was suggested to be associated with partial BD, and not with a 45–50% reduction of biotinidase activity, as expected. In the present study, among the seven cases of disagreement between the observed and expected biochemical phenotype, we highlight patient 10 because her genotype p.D444H/c.595G > A (p.V199M) and p.V199M/p.V199M have previously been reported in patients with residual biotinidase activity levels of 32% and 11.5% respectively [26], and it is possible that the p.V199M variant is not as damaging as other profound BD alleles. Furthermore, patient 31, from Northeast Brazil, and patients 32, 34 and 35, from the Southeast region, underwent testing at a laboratory in the South region, so their biochemical test results may have been artifactually low due to time constraints or transportation of samples to the laboratory under inadequate conditions, as suggested by Neto et al. (2004) [18]. However, we cannot rule out the presence of changes in a region of the *BTD* gene not covered by our analyses causing decreased enzyme activity.

In three patients, we were unable to define the *cis*/*trans* status of variant p.D444H in relation to the other variant found. On the basis of enzyme activity, *trans* status is most probable for patient 16. For patient 17, who presented with a biotinidase level suggestive of heterozygosity (but very close to the upper limits of partial DB), only gDNA from her mother, who also presented low biotinidase activity in a dried spot blood sample (data not shown), was available for analysis; as she was found to be a carrier of both p.D444H and c.643C > T (p.L251F) variants, the patient can be heterozygous if both variants were inherited on the maternal side (if they were *in cis*), or have partial BD if p.D444H was inherited from the father and only p.L215F from the mother (in this case, the mother should also exhibit partial DB). Similarly, both parents of patient 18, who is heterozygous for variants p.D444H and c.1629C > A (p.D543E), are heterozygous for p.D444H, but the father is also a carrier of p.D543E; therefore, the patient can be heterozygous if p.D444H and p.D543 are in *cis* in the paternal allele, or can have partial DB if they are in *trans* (in which case the father should also exhibit partial DB). A new enzyme activity test in the child and testing of parents (or even analysis of grandparent DNA) could help characterize BD in this case.

Although the enzyme activity of symptomatic patients 32, 34 and 35 was not consistent with profound or partial BD, biotin treatment was started after other genetic conditions had been ruled out, as the patients’ clinical manifestations are consistent with BD and biotin has a good safety profile. Unfortunately, we are not aware whether there was any improvement with treatment. These patients may represent cases of delayed-onset BD, but the absence of variants in exons 2–4 of the *BTD* gene suggest they do not have BD. Patient 33 is also very interesting, since his biotinidase activity levels were very low on neonatal screening, and gradually approached the normal range thereafter, as would be expected to his genotype. We believe the wide variation in biotinidase levels presented by this patient is mainly explained by premature birth and neonatal jaundice. However, as the first test was performed when he was only 5 days old and the last at age 1 year, some of this variation could also be due to increasing age.

On the basis of the allele frequency of novel variants p.L40P and p.C160Y in controls and of the *in silico* prediction of pathogenicity, and taking observed enzyme activity into account, these alleles were considered probably damaging, and patients 36 and 37, who are compound heterozygous for the p.D444H variant, probably have partial BD. Variant p.L40P does not affect the peptide portion that constitutes the mature protein, but affects the signal peptide, which may impair targeting of the enzyme to secretory vesicles, thus resulting in reduced enzyme activity in plasma. Alleles associated with profound BD which affect this region have been described elsewhere [27],[28]. The effect of variant p.D222N on biotinidase function could not be established clearly from bioinformatics, as we used two different software programs which presented contradictory conclusions regarding this variant. However, the absence of this variant in controls suggests it is probably damaging.

Regarding the other two nonpathogenic polymorphic variants found in controls, c.1171C > T or p.P391S (rs35034250) and c.1413 T > C or p.C471C (rs3817641), both were found at frequencies similar to those reported in populations of USA and African origin respectively (available at: http://www.ncbi.nlm.nih.gov/snp/).

## Conclusion

Genotyping of Brazilian patients with reduced biotinidase activity broadened current knowledge on the allelic heterogeneity of BD. Furthermore, the high frequency of the p.D444H variant in patients and controls suggest that the incidence of partial BD in Brazil may be higher than worldwide estimates. When enzyme activity approaches the border between partial BD and heterozygosity, sequencing of the *BTD* gene (starting with analysis of exons 2 and 4) appears to be the most appropriate technique to elucidate the diagnosis in this population. We suggest that parental DNA analysis be carried out whenever the p.D444H variant and another variant are detected in a BD patient.

## Abbreviations

BD: Biotinidase deficiency

NB: Newborns

PCR: Polymerase chain reaction

PEG: Polyethylene glycol

RFLP: Restriction fragment length polymorphism

F: Female

M: Male

NS: Neonatal screening

S: Symptoms

Hz: Heterozygosity

N/A: Not available

N: Normal

D: Days

M: Months

Y: Years

FIPE: *Fundo de Incentivo à Pesquisa e Eventos*

HCPA: *Hospital de Clínicas de Porto Alegre*

CNPq: National Council for Scientific and Technological Development

FAPERGS: Rio Grande do Sul Research Foundation

## Competing interests

The authors declare that they have no competing interests.

## Authors’ contributions

IVDS conceived of the study, participated in its design and coordination and drafted the manuscript. TB collected clinical information, carried out the molecular analysis, and drafted the manuscript. FS assisted in the interpretation of results. SL participated in the design of the study. The other authors contributed in recruiting participants, collecting samples and data. All authors read and approved the final manuscript.
